# Surface EMG pattern recognition for real-time control of a wrist exoskeleton

**DOI:** 10.1186/1475-925X-9-41

**Published:** 2010-08-26

**Authors:** Zeeshan O Khokhar, Zhen G Xiao, Carlo Menon

**Affiliations:** 1 MENRVA Group, School of Engineering Science, Faculty of Applied Science, Simon Fraser University, 8888 University Drive, Burnaby, BC, V5A 1S6, Canada

## Abstract

**Background:**

Surface electromyography (sEMG) signals have been used in numerous studies for the classification of hand gestures and movements and successfully implemented in the position control of different prosthetic hands for amputees. sEMG could also potentially be used for controlling wearable devices which could assist persons with reduced muscle mass, such as those suffering from sarcopenia. While using sEMG for position control, estimation of the intended torque of the user could also provide sufficient information for an effective force control of the hand prosthesis or assistive device. This paper presents the use of pattern recognition to estimate the torque applied by a human wrist and its real-time implementation to control a novel two degree of freedom wrist exoskeleton prototype (WEP), which was specifically developed for this work.

**Methods:**

Both sEMG data from four muscles of the forearm and wrist torque were collected from eight volunteers by using a custom-made testing rig. The features that were extracted from the sEMG signals included root mean square (rms) EMG amplitude, autoregressive (AR) model coefficients and waveform length. Support Vector Machines (SVM) was employed to extract classes of different force intensity from the sEMG signals. After assessing the off-line performance of the used classification technique, the WEP was used to validate in real-time the proposed classification scheme.

**Results:**

The data gathered from the volunteers were divided into two sets, one with nineteen classes and the second with thirteen classes. Each set of data was further divided into training and testing data. It was observed that the average testing accuracy in the case of nineteen classes was about 88% whereas the average accuracy in the case of thirteen classes reached about 96%. Classification and control algorithm implemented in the WEP was executed in less than 125 ms.

**Conclusions:**

The results of this study showed that classification of EMG signals by separating different levels of torque is possible for wrist motion and the use of only four EMG channels is suitable. The study also showed that SVM classification technique is suitable for real-time classification of sEMG signals and can be effectively implemented for controlling an exoskeleton device for assisting the wrist.

## Background

sEMG can provide information regarding the neural activation of muscles, which can be used to estimate the intention of the person and also identify potential neuromuscular disorders [1]. The use of sEMG signals has been explored for different applications. One of the applications of sEMG signals is in regards to rehabilitation through robotic devices. It has been proposed that sEMG signals can be used to quantify the assessment of hand functions [2] and robotic devices can be used to provide an assistive force as a compensation for hand movement [3]. Combining sEMG signals with robotic therapy can optimize the coordination of motor commands and actual movement [4-6]. Another application of EMG signals is in the control of prosthetic hands. Numerous prosthetic hands have been prototyped, including the CyberHand [7] and SmartHand [8], and some have also been commercialized, including the iLimb [9] and the Otto Bock's SensorHand Speed [10]. In these research and development efforts, the goal was to obtain a lightweight and dexterous prosthetic hand that could perform movements similar to a human hand. A crucial aspect towards an effective use of these prosthetic hands is their intuitive control, which could be achieved through detection and interpretation of the user's neurological activity to be detected, for example, through sEMG electrodes. Whether used for controlling an assistive, rehabilitative or prosthetic device, the basic challenge is to be able to process sEMG signals and identify the intention of the user. Different studies have been performed to tackle this challenge by using different pattern recognition methods [11-28].

The analysis of pattern recognition in sEMG mainly consists of two steps, namely feature extraction and classification. Feature extraction is the dimensionality reduction of the raw sEMG input to form a feature vector - the accuracy of the pattern classification system almost entirely depends on the choice of these features [11]. Features cannot be extracted from the individual samples as the structural detail of the signal will be lost and hence the features need to be calculated by segmenting the raw sEMG signal and calculating a set of features from each segment [11]. Researchers have experimented with the length of the segment and the constraint in the length mainly derives from the specific real-time implementation. A delay of 200~300 ms interval is the clinically recognized maximum delay tolerated by the users [29]. A suitable delay for the controller to generate a control command should therefore be between 100~125 ms [30]. Different features have been used in pattern recognition involving both time domain and time-frequency domain features. Some of these include mean absolute value [11,12,15-17], zero crossings (ZC) [11,12,15-17], slope sign changes (SSC) [11,12,15,16], autoregressive (AR) model coefficients [12,15,18-20], cepstrum coefficients [19], waveform length (WL) [11,12,16,17] and wavelet packet transform[13-15].

As regards to classification, it can be defined as the process of assigning one of *K *discrete classes to an input vector ***x ***[31]. Numerous studies have been done to classify the features extracted from the sEMG like neural networks [11,20,21], bayesian classifier [24], linear discriminant analysis [16,23], hidden markov model [26], multilayer perceptron [13,14,23], fuzzy classifier [15,17-19], gaussian mixture model [12] and support vector machines (SVM) [21,22,27,28].

Feature extraction and classification methods were primarily used in previous research studies to identify the orientation of the hand without considering the amount of force the user was applying. In the use of advanced hand prostheses, it would however be beneficial having control over the amount of force a person intends to apply and, for assistive devices, force control would indeed be necessary. Castellini *et al*. [21] successfully controlled the amount of force applied by the fingers in different types of grasp so that the user could apply a different amount of force for holding, for example, a hammer or an egg [21].

**Figure 1:**
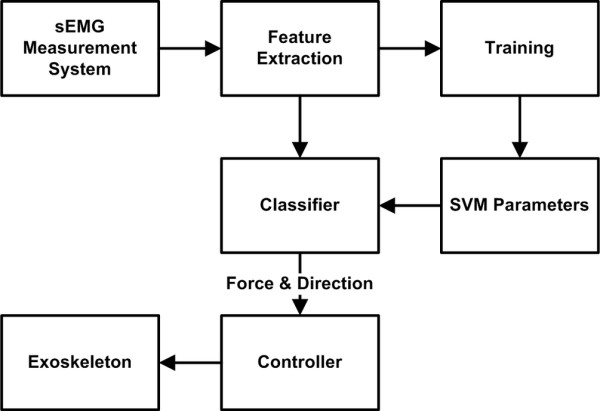
**Overview of the real-time classification system**.

In this paper, we focus on the identification of both the direction and intensity of the torque applied by the wrist - a particular direction and a particular force range defines a class. We have experimented with two sets of data involving nineteen and thirteen classes. A WEP with two degrees of freedom was developed to test the classification system in real time. Figure 1 shows the block diagram of the classification system. The sEMG signals were measured by using a commercial measurement unit and after some processing, as explained in the following methods section, features including sEMG rms value, AR model coefficients and waveform length, were extracted. SVM was used as a classifier as it is suitable for real-time applications. The result of classification was fed to a custom-designed controller, which controlled the force and direction of the WEP actuators.

### Support Vector Machines

Support Vector Machines [32] is a classification technique based on maximizing the margin between a data set and the hyper plane separating two data sets. In a general form, SVM requires solving the following optimization problem

(1) $$\begin{array}{lll} \min & \frac{1}{2}{\left\|w\right\|}^{2}+C\sum_{n=1}^{N}\xi_{n} & \\ \text{subject to} & \begin{array}{l} {\text{t}}_{\text{n}}y(x_{n})\geq 1-\xi_{n},\\ \xi_{n}\geq 0 \end{array} & n=1,...,N \end{array}$$

where *N *is the number of data points, ***x***_*n *_is the vector representing a data point, *t_n _*is the label associated with a data point, *y *is the learned model, ***w ***is the vector representing adaptive model parameters, *ξ*_*n *_is the slack variable and *C > 0 *is the penalty factor. Although SVM linearly separates two data sets, different researchers have introduced the use of kernels in the algorithm to extend it for non-linear separation without much increase in computational complexity. Some of the well-known kernels include polynomial, radial basis, Gaussian and sigmoid. SVM, which is a two class separation technique, has also been extended for multiclass classification. This is done by splitting a single multi-class problem to multiple binary classification problems. The two most common methods are one-versus-one and one-versus-all, whose details are presented in [33]. An important property of SVM is that the model parameter estimation corresponds to a convex optimization problem meaning that any local solution will be a global optimum [33]. SVM also has a high generalization ability making it suitable for unseen data; it has recently been successfully applied to bio-information signals for pattern recognition [34-37].

## Methods

### EMG electrode placement and data acquisition

**Figure 2:**
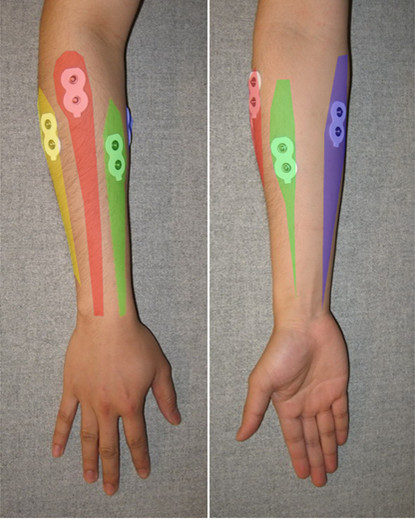
**Position of muscles of the forearm**. ED is shown in red, ECR in yellow, PL in green and FCU in purple color.

Several forearm muscles contribute to the movement of the wrist, details of which can be found in [38]. Four forearm muscles were identified as suitable candidates for classification through a trade-off experimental procedure. The four selected muscles were Flexor Carpi Ulnaris (FCU), Palmaris Longus (PL), Extensor Digitorum (ED) and Extensor Carpi Radialis (ECR). FCU assists in wrist flexion with ulnar deviation, PL assists in wrist flexion, ED assists in extension of four fingers and aids in extension of the wrist and ECR assists in extension and radial abduction of the wrist. The approximate position of these muscles is shown in Figure 2.

Reliable sEMG data acquisition is necessary before extracting features for classification. Numerous factors affect the quality of sEMG acquisition such as inherent noise in the electronic equipment, ambient noise in the surrounding atmosphere, motion artefacts and poor contact with skin. The first three factors are dependent on the sEMG acquisition system used and, to reduce the effects of these, a commercial sEMG system from Noraxon (Myosystem 1400L) was used. In order to have a good skin contact with the electrodes, the guidelines of the surface electromyography for the non-invasive assessment of muscles (SENIAM) project [39] were followed. The skin of the volunteer was shaved and an alcohol swab was used to clean the skin. The electrodes were placed at the desired locations after the skin dried. We used AgCl gel dual electrodes from Noraxon, which contains two electrodes at a recommended distance. The usable energy in an EMG signal lies in the range of 0-500 Hz [40] and therefore the acquired sEMG signal was digitized at 1024 samples per second using a data acquisition card from National Instruments (NI USB-6289) and stored on a computer by the LabVIEW software.

### Data collection setup and protocol

**Figure 3:**
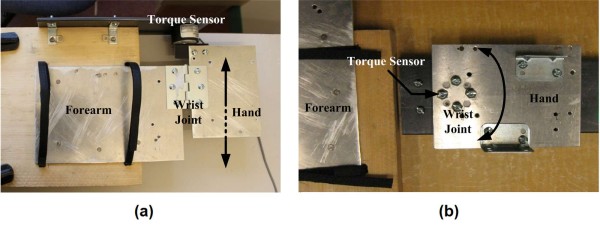
**Testing rigs to measure wrist torque**. (a) Rig to measure torque during wrist flexion/extension; and (b) Rig to measure torque during wrist ulnar/radial deviation.

A total of eight volunteers, who signed an informed consent form (project approved by the Office of Research Ethics, Simon Fraser University; Reference # 2009s0304), participated in the current study. Two testing rigs were built to record the direction and level of torque applied by the wrist. The first rig was designed to record the level of torque for flexion/extension of the wrist (see Figure 3(a)) and the second rig to record the level of torque for ulnar/radial deviation of the wrist (see Figure 3(b)). Both rigs consisted of two separate sheets of aluminium connected together with a reaction torque sensor (Transducer Techniques TRT-100). The forearm rested on one plate and the hand rested on the second such that the torque sensor read the torque produced at the wrist joint. An application was developed using LabVIEW software to simultaneously acquire both the sEMG signals and the torque readings. Each volunteer followed the twelve protocols summarized in Table 1. Protocols 1, 2, 5 and 6 were used to record the maximum torque produced by the user in each direction and this was designated as the maximum voluntary contraction (MVC). A visual bar graph was represented on the screen of a monitor to provide a visual feedback of the produced wrist torque in real-time - this feature was needed especially to complete protocols 3, 4, 7 and 8, which were used to generate data for the formation of the classes. All the protocols listed in Table 1 never exceeds 50% of the MVC because studies have shown that in order to avoid upper extremity musculoskeletal injuries force should not exceed 40-50% of the maximum [41].

**Table 1:** Protocol Information

Protocol Number	Action	Number of Repetition
1	Wrist flexion with maximum torque	3

2	Wrist extension with maximum torque	3

3	Wrist flexion: start from rest and increase torque by 10% of MVC after every 10 seconds until 50% of MVC is applied	3

4	Wrist flexion: start from 50% of MVC and decrease torque by 10% after every 10 seconds until no torque is applied	3

5	Wrist extension: start from rest and increase torque by 10% of MVC after every 10 seconds until 50% of MVC is applied	3

6	Wrist extension: start from 50% of MVC and decrease torque by 10% after every 10 seconds until no torque is applied	3

7	Wrist ulnar deviation with maximum torque	3

8	Wrist radial deviation with maximum torque	3

9	Wrist ulnar deviation: start from rest and increase torque by 10% of MVC after every 10 seconds until 40% of MVC is applied	3

10	Wrist ulnar deviation: start from 40% of MVC and decrease torque by 10% after every 10 seconds until no torque is applied	3

11	Wrist radial deviation: start from rest and increase torque by 10% of MVC after every 10 seconds until 40% of MVC is applied	3

12	Wrist radial deviation: start from 40% of MVC and decrease torque by 10% after every 10 seconds until no torque is applied	3

### Feature extraction and classification

After the data collection, the acquired samples were converted into features that could be used for classification. Matlab software was used to extract and classify the features using the recorded sEMG signals. Features were extracted from the samples by segmenting the signal into 250 ms intervals corresponding to 256 samples in each segment. A single feature was calculated from each segment and the segment window was incremented by 125 ms (128 samples) for the next feature. This scheme ensured that a control command could be generated within 250 ms from the instant the user's intention was given. Three kinds of features were extracted from each segment namely EMG rms value, AR model coefficients and WL. The EMG rms value, *r_k_*, is computed as:

(2) $$r_{k}=\sqrt{\frac{\sum_{i=1}^{N}x_{i}^{2}}{N}}$$

where *x_i _*is the value of the *i^th ^*sample in the *k^th ^*segment and *N *is the number of samples, which in our case is 256.

AR models are constructed using a recursive filter. This filter predicts the current value based on the previous output values of the filter. The current value *y(t) *can be computed as:

(3) $$y(t)=\sum_{i=1}^{m}a_{i}y(t-i)+\varepsilon (t)$$

where *a_i _*are the model coefficients, *m *is the order of the model and *ε *is the output error. We used the AR model coefficients as the features with a model order of four, which is adequate for modelling EMG signals [42], thus generating four features for each channel of sEMG.

The third kind of extracted feature was the waveform length, which provided a measure of the waveform complexity in each segment. The waveform length *l *can be mathematically represented as:

(4) $$l=\sum_{k-1}^{N}\left|\Delta x_{k}\right|=\sum_{k-1}^{N}\left|x_{k}-x_{k-1}\right|$$

We used four channels of sEMG data, which therefore provided 24 features per segment. As regards to classification, the LibSVM tool [43] was used in the Matlab environment. LibSVM has an implementation for multi class SVM using one-versus-one strategy and provides a choice of four basic kernels namely linear, polynomial, radial basis function (RBF) and sigmoid. As discussed in [44,45], RBF is in general a reasonable first choice as it maps the samples nonlinearly and has few numbers of hyperparameters reducing the complexity of model selection. For this reason, RBF was selected as a kernel in the SVM:

(5) $$K(x_{i},x_{j})=\exp(-\gamma {\left\|x_{i}-x_{j}\right\|}^{2}),\;\gamma >0$$

We used eight fold cross validation along with grid search to find the optimal parameters for *C *and *γ*.

The sEMG data gathered from the volunteers was analyzed in two configurations. The first configuration consisted of nineteen classes and the second one used thirteen classes. The purpose of using two different configurations was to obtain preliminary results enabling a trade-off between the accuracy of the classifier and the smoothness of the torque provided by the assistive device. Six seconds of data per iteration per protocol was extracted for each class, which provided 5358 data segments per class. Out of these, 4788 data segments were used as training data and 570 data segments were used as testing data. Table 2 specifies the 19 classes used for the first configuration. For the second configuration (13 classes), class 3, 5, 8, 10, 13 and 17 were removed. The division of classes is at particular force level but the SVM classifier works on maximizing the margin between the adjacent classes meaning that in an ideal case, the boundary between two adjacent classes will be exactly in the middle such that a flexion with 15% MVC to 25% MVC will belong to class 3. In practical scenarios these boundary levels may differ based on how accurately the volunteer was able to follow the training protocol.

**Table 2:** Actions for different classes

Class No	Associated action
1	Resting position

2	Flexion with 10% of MVC torque

3	Flexion with 20% of MVC torque

4	Flexion with 30% of MVC torque

5	Flexion with 40% of MVC torque

6	Flexion with 50% of MVC torque

7	Extension with 10% of MVC torque

8	Extension with 20% of MVC torque

9	Extension with 30% of MVC torque

10	Extension with 40% of MVC torque

11	Extension with 50% of MVC torque

12	Ulnar deviation with 10% of MVC torque

13	Ulnar deviation with 20% of MVC torque

14	Ulnar deviation with 30% of MVC torque

15	Ulnar deviation with 40% of MVC torque

16	Radial deviation with 10% of MVC torque

17	Radial deviation with 20% of MVC torque

18	Radial deviation with 30% of MVC torque

19	Radial deviation with 40% of MVC torque

### Mechanical design and control of exoskeleton

**Figure 4:**
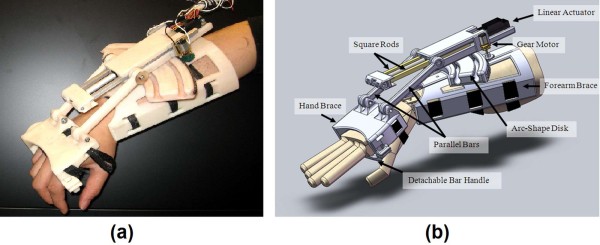
**Wrist exoskeleton prototype (WEP)**. (a) Picture of WEP; and (b) CAD drawing of WEP.

To test the real-time classification system, the WEP was developed; a picture along with its CAD representation is shown in Figure 4. The WEP is a preliminary prototype, which was designed to be portable and lightweight for potentially being used in the future for rehabilitation or assistance. The WEP is designed to assist the wrist both in flexion/extension and ulnar/radial deviation. The WEP structure is made of ABS plastic and mainly consists of two braces for the forearm and the hand. The overall size of the forearm brace is 19.7 cm × 16.8 cm × 11.1 cm and the size of the hand brace is 7.0 cm × 12.4 cm × 6.0 cm. With a total weight of about 500 g including actuators, the WEP is easy to carry and allows the user to potentially wear it in different environments. To prevent possible injures, the WEP motion was mechanically restricted to 60 degree for wrist flexion, 60 degree for extension, 30 degree for radial deviation and 30 degree for ulnar deviation. Further constraints can be applied for different users.

The flexion/extension motion is provided by a linear actuator, having 10 cm stroke length (Firgelli L12-100-210-12-P), which is fixed to a moveable housing coupled to an arc-shaped disk of the forearm brace, as shown in Figure 4. The head of the linear actuator is connected to a block having two aluminium square rod extensions used to improve the stiffness of the WEP during actuation. Two parallel bars are attached to connect the aluminium extensions with the hand brace through revolute joints. The linear actuator is able to deliver about 2.2 Nm of torque to the wrist over the entire flexion-extension range of motion when supplied with 12 V.

To control the ulnar/radial deviation of the wrist, a gear motor (Pololu 298:1 micro metal gear motor) is attached to a side of the linear actuator housing, and coupled to the outer side of the arc-shape disk with a spur gear. The ratio between the arc-shape disk's radius and the one of the spur gear is 15:1; thus, the torque generated by the gear motor is amplified by a factor of 15 at the wrist joint. With the use of the Pololu gear motor, a maximum torque of 5.4 Nm can be applied at the wrist joint for ulnar/radial deviation.

**Figure 5:**
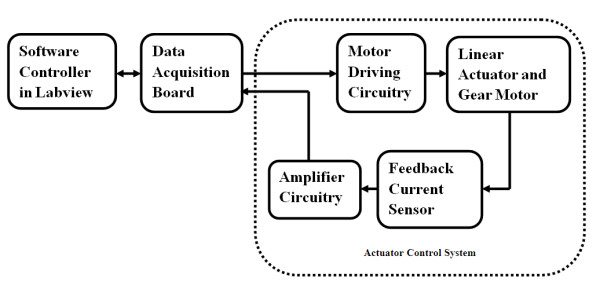
**Block diagram for actuator control system**.

A simplified force-feedback control system is implemented to operate the WEP. The control system consists of six different functional blocks, which are shown in Figure 5. A PID control algorithm is used for controlling the current through the actuators by varying the duty cycle of two 20 kHz Pluse Width Moduated (PWM) signals. These signals reach a motor driving circuity through a data acquisition board (National Instruments USB-6289) to control the motors, while the current of the motors are read by a current sensor and then amplified to serve as feedback data for the force control.

### Real-time experimental setup

**Figure 6:**
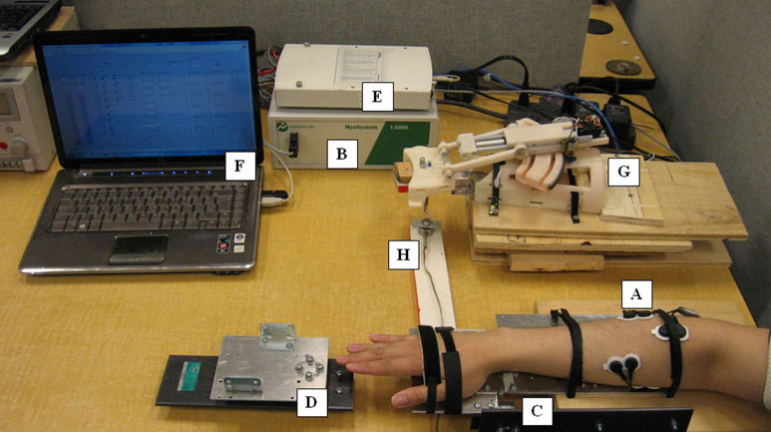
**Real-time experiment setup**. (A) sEMG leads, (B) sEMG measuring device, (C) torque measuring device for wrist flexion-extension, (D) torque measuring device for wrist ulnar-radial deviation, (E) data acquisition board, (F) classifier and force controller in LabVIEW, (G) WEP, and (H) force sensor.

**Figure 7:**
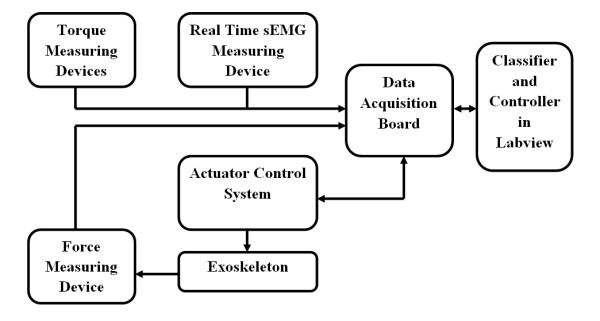
**Block diagram of the experiment setup**.

To test the performance of the system in real-time, a LabVIEW application was developed. This application implemented LibSVM in the LabVIEW environment along with the feature extraction techniques and control of the exoskeleton. A picture of the experimental setup is shown in Figure 6. The setup included the custom rigs for measuring the torque produced by the wrist of the volunteer during flexion/extension and ulnar/radial deviation, sEMG acquisition system (Noraxon Myosystem 1400L), data acquisition card (National Instruments USB-6289), laptop running the LabVIEW application, WEP secured on a wooden palm attached to a platform and a force sensor (Futek LCM300) connected to the wooden palm to record the force produced by the WEP. A block diagram representing the interconnection between the different components is shown in Figure 7.

The real-time experiment consisted of two steps: training and testing. During the first step, the volunteer was asked to place the right forearm on the custom made rig, which indicated the torque applied by the user in real-time. The sEMG acquisition system, presented in the data acquisition section of this paper, was used. The torque and EMG data were digitalized at a frequency of 1024 samples per second. The volunteer applied the torque according to the proposed protocol (see Table 1) and 13 classes were trained. In the second step, the volunteer applied different torques by using the same setup and the LabVIEW application predicted the wrist output through the only real time sEMG input and provided the control signal to actuate the WEP, which applied torque corresponding to the identified class.

### Wrist assistance: proof of concept

**Figure 8:**
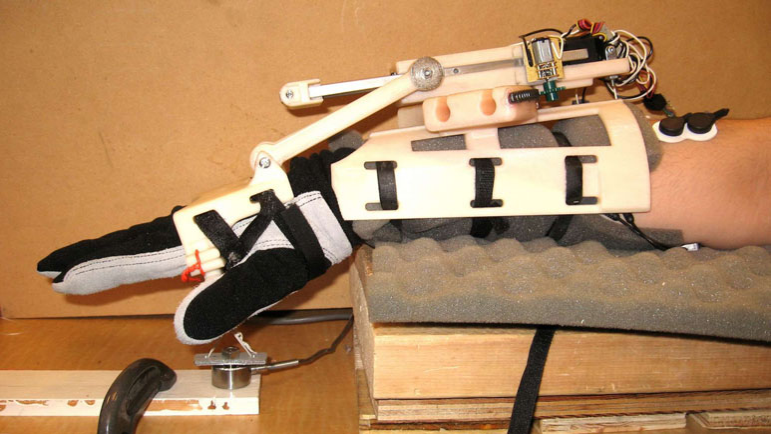
**Experiment setup for amplification of wrist extension**.

To demonstrate the potential ability of using the WEP as an assistive device with the proposed classification method, an experiment was conducted. A volunteer was asked to wear a glove (used for ensuring safety during testing), the WEP, four set of electrodes attached to the FCU, PL, ED and ECR, and to place the forearm onto a wooden platform as shown in Figure 8. A force sensor was attached to the bar handle of the WEP to record the isometric force during the extension of the wrist (see Figure 8).

The purpose of the test was to enable a comparison between the rms values of the sEMG with and without the WEP assistance. The overall experiment consisted of three steps: (1) training for the classification system, (2) wrist extension with assistance from the WEP and (3) wrist extension without assistance. During the training step, the parallel bars of the WEP were detached from the hand brace so that the wrist was not constrained and the force sensor could read the applied force. The classification system was then trained for four classes corresponding to rest, 10% of MVC, 20% of MVC and 30% of MVC. In the next step, the parallel bars of the WEP were attached back to the WEP to assist the wrist extension. The volunteer was asked to pull against the force sensor, and maintain a strength that corresponded to a particular class for a short period - the WEP was expected to assist the wrist extension. In the last step, the parallel bars of the WEP were detached again from the hand brace to remove the assistance. The volunteer was subsequently asked to pull against the force sensor to a force level that was achieved with assistance, and maintain that force level for a short period of time - visual feedback of the applied force was provided to the volunteer.

## Results and Discussion

### Offline experiments

As mentioned earlier, we analyzed the data in two configurations. The configuration with 19 classes consisted of a training feature vector of size 4788 × 24. After cross validation and grid search to find the optimal parameters, the prediction was tested by using a test feature vector of size 570 × 24. The average accuracy, which was computed by taking into account both false negatives and false positives as proposed in [46], resulted to be equal to 88.2%. Table 3 summarizes the results of classification on each individual volunteer.

**Table 3:** Classification results with 19 classes

Volunteers	C	γ	Cross Validation Accuracy (%)	Testing Accuracy (%)
Volunteer # 1	45	1	94.05	90.8621

Volunteer # 2	60	0.8	94.07	92

Volunteer # 3	85	1	90.24	85.67

Volunteer # 4	90	0.9	91.94	86.5

Volunteer # 5	75	1	88.77	86

Volunteer # 6	85	1	88.11	84

Volunteer # 7	75	1	90.58	87

Volunteer # 8	90	0.7	94.26	93.57

**Mean**			91.5025	88.20026

**Standard Deviation**			2.458151	3.455318

Results obtained for classification accuracy in volunteers who had greater MVC and those who could maintain a torque level with little variation were much better than the rest. Also, most of the errors were due to a class misclassified in an adjacent class. The average accuracy for the eight volunteers neglecting misclassification in adjacent classes reached up to 99.99%. This suggests that the cause of lower accuracy is the small separation between torque levels; to evaluate the trade-off between smoothness of torque and average accuracy of the classifier, the second configuration was analyzed.

This second configuration consisted of 13 classes with a training feature vector of size 3276 × 24 and a testing feature vector of size 390 × 24. Using the same 8 fold cross validation and grid search, it was observed that the average accuracy increased to 96.52%. The classification accuracies for individual volunteers are shown in Table 4. The accuracy reached 99.72% in the case of the first volunteer.

**Table 4:** Classification results with 13 classes

Volunteer	C	γ	Cross Validation Accuracy (%)	Testing Accuracy (%)
Volunteer # 1	50	0.7	99.72	97.95

Volunteer # 2	60	1	98.61	98.57

Volunteer # 3	80	1	98.1	94.76

Volunteer # 4	90	0.9	97.39	94.05

Volunteer # 5	75	1	95.83	94.76

Volunteer # 6	70	1	96.8	96.19

Volunteer # 7	80	0.9	97.71	96.43

Volunteer # 8	90	1	99.58	99.47

**Mean**			97.97	96.52

**Standard Deviation**			1.33	1.98

Tables 3 and 4 show that, as expected, classification accuracy decreased when the number of classes increased but still good results were obtained with the highest number of classes. Depending on the needs of specific future practical applications, which could have different requirements on the smoothness of the output torque of the assistive device or high precision in the identification of the user intention, the number of classes could therefore be selected appropriately and could be between 13 and 19 classes.

### Real-time experiments

The performance of the classification system in real-time was studied by controlling the WEP by the sEMG signals of the forearm. A control signal was sequentially generated by the system after every 125 ms and the sEMG signals from the data acquisition card was acquired every 125 ms ensuring that the total response time for the system was less than 250 ms. These delays are acceptable for real-time systems as indicated in [29,30].

**Figure 9:**
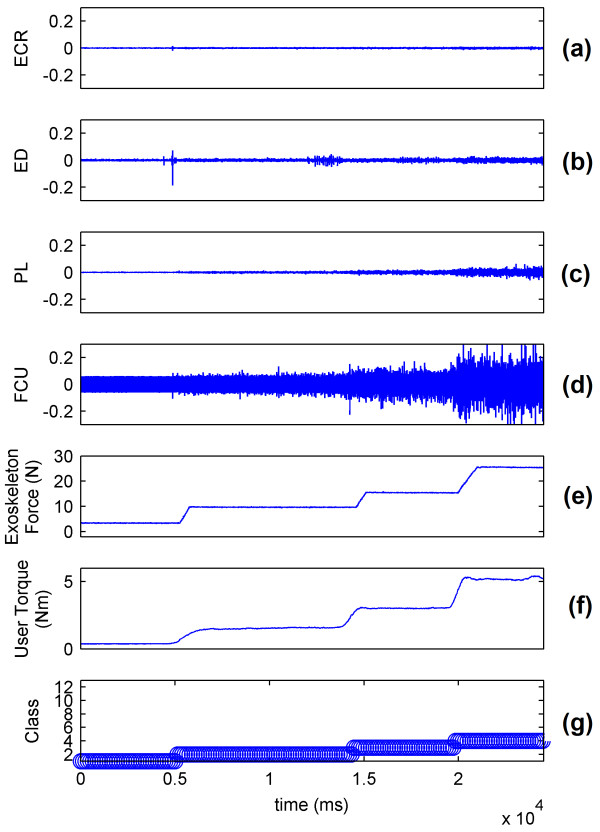
**System performance for wrist flexion**. (a) ECR muscle activation; (b) ED muscle activation; (c) PL muscle activation; (d) FCU muscle activation; (e) Force applied by exoskeleton; (f) Torque applied by the wrist of volunteer; and (g) Identified class by the system.

**Figure 10:**
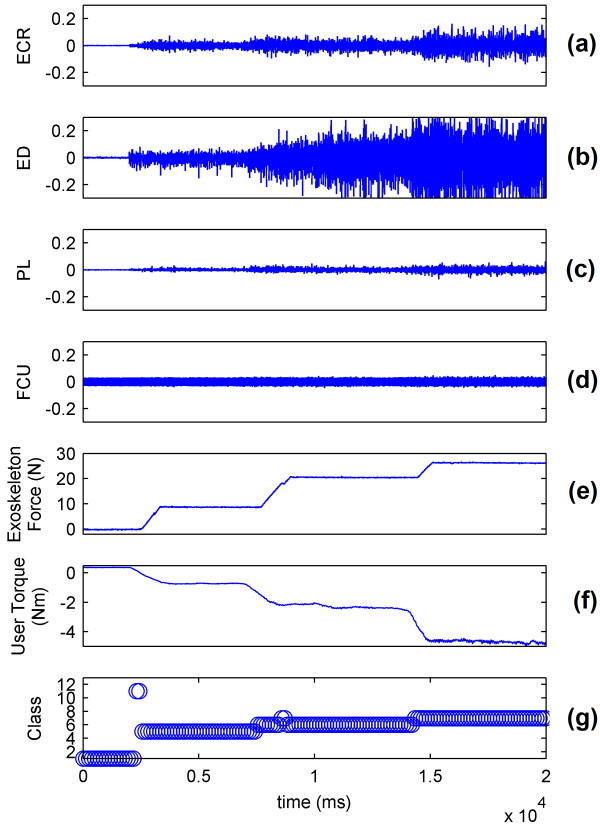
**System performance for wrist extension**. (a) ECR muscle activation; (b) ED muscle activation; (c)PL muscle activation; (d) FCU muscle activation; (e) Force applied by exoskeleton; (f) Torque applied by the wrist of volunteer; and (g) Identified class by the system.

**Figure 11:**
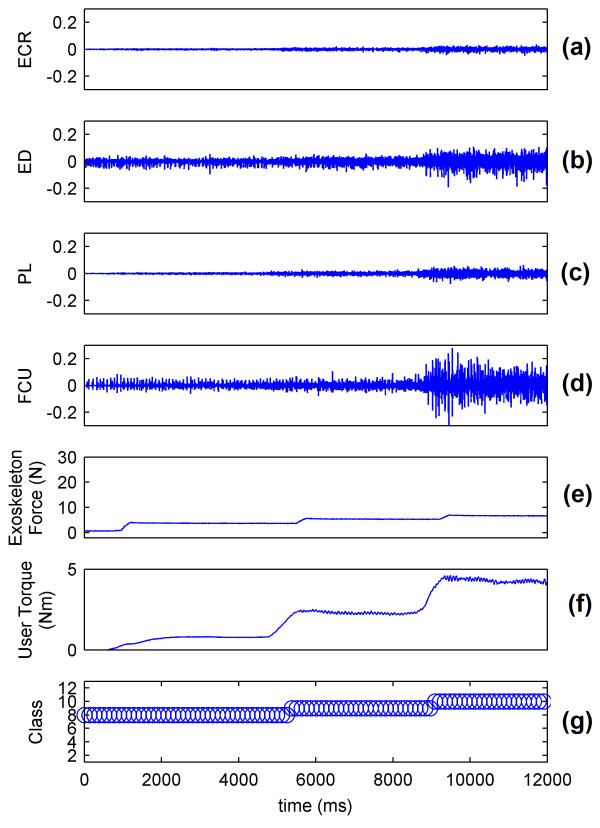
**System performance for wrist radial deviation**. (a) ECR muscle activation; (b) ED muscle activation; (c) PL muscle activation; (d) FCU muscle activation; (e) Force applied by exoskeleton; (f) Torque applied by the wrist of volunteer; and (g) Identified class by the system.

**Figure 12:**
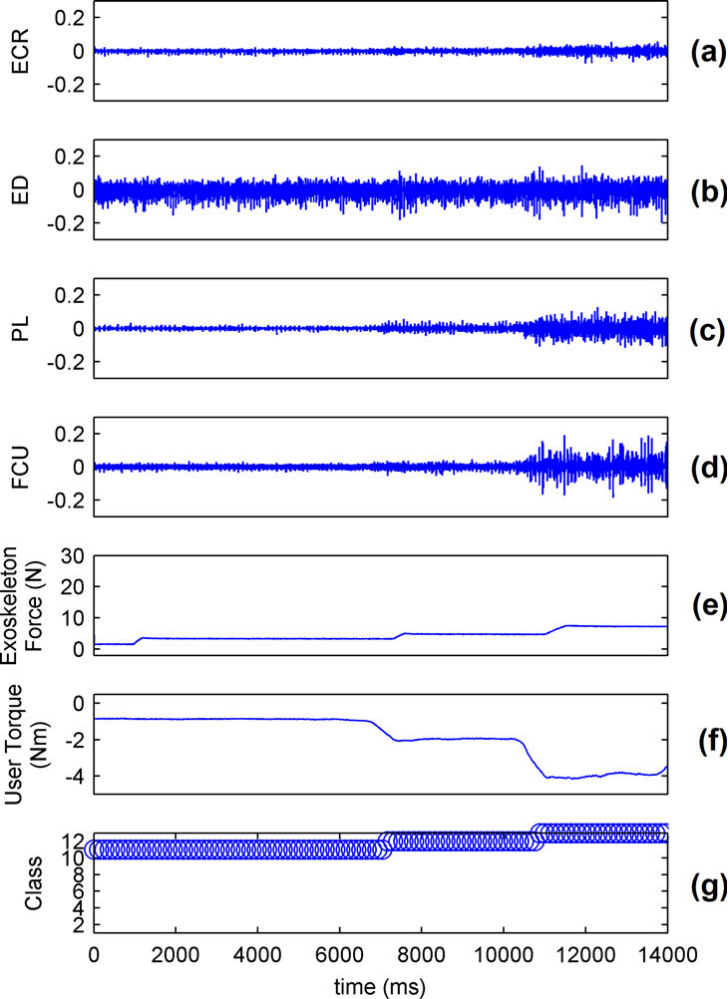
**System performance for wrist ulnar deviation**. (a) ECR muscle activation; (b)ED muscle activation; (c)PL muscle activation; (d) FCU muscle activation; (e) Force applied by exoskeleton; (f) Torque applied by the wrist of volunteer; (g) Identified class by the system.

The sEMG signals of the wrist show that the muscle mainly responsible for flexion is the FCU (Figure 9(a), (b), (c) and 9(d)). The real-time system predicts the intention of the volunteer and controls the WEP to apply forces corresponding to the applied torque (Figure 9(e) and 9(f)). The decision to control the force of the WEP is determined by the identified class (Figure 9(g)). The results for wrist extension, radial deviation and ulnar deviation are respectively presented in Figures 10, 11 and 12.

Figures 9, 10, 11 and 12 show that the classification system predicts the torque and direction of the user with a good accuracy. The few errors observable in the system also indicate that the misclassified points lie in the adjacent class meaning only the level of torque is incorrectly predicted and not the direction of movement. It is to be noted that the delay in reaching a particular force value for the exoskeleton is due to the response time of the exoskeleton and not to the response time of the classification system.

### WEP as an assistive device

**Figure 13:**
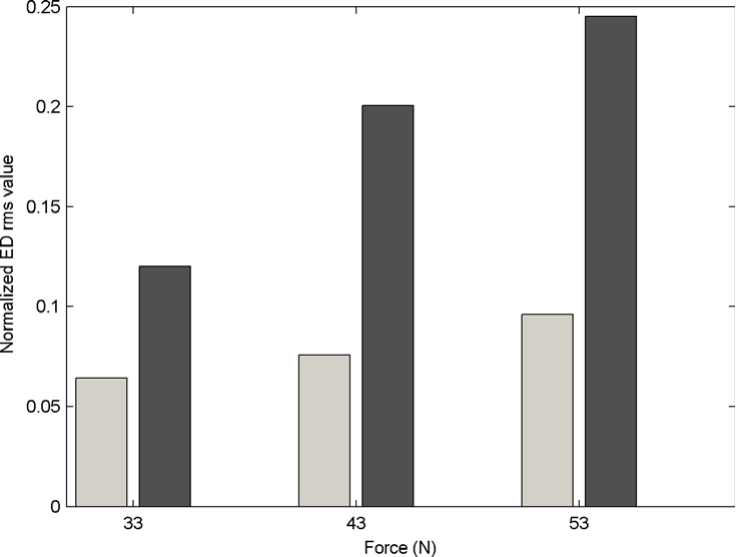
**Comparison of ED rms value with and without WEP**.

Figure 13 shows the sEMG rms value over a period of one second for the ED muscle when applying approximately 33, 43 and 53 Newton of force in both cases in which the volunteer was and was not wearing the WEP. Figure 13 shows that the ED rms value was considerably less when the WED was worn, thus proving the potential assistive features of the WED and real-time classification system. It should be noted that the force level applied by the WEP can be set to be a specific percentage identified by the user - the WEP could therefore assist the user by augmenting a percentage of her/his wrist torque.

## Conclusions

This paper explores the possibility of using sEMG signals to control the torque applied by the wrist along with direction of motion. Data was gathered from four forearm muscles during isometric movements of the wrist by using a commercial EMG measurement system and a custom designed rig. sEMG signal rms values, AR model coefficients and waveform length were used to extract features and SVM was used to classify torque of the wrist both into 19 and 13 classes. The average accuracy for 19 classes was about 88% and for 13 classes was 96%. According to the needs of future specific applications, any number of classes in between these two could therefore be potentially suitable. A wrist exoskeleton prototype was developed to study the performance of the real-time system and a proof of concept for the use of WEP as an assistive device was presented. The system was able to respond to user's intention within 250 ms proving that SVM is a suitable technique to be used in real-time sEMG recognition system. The classification system investigated in this study used isometric wrist measurements to simplify the analysis of the investigated problem. Future work will investigate the feasibility of combining force control during dynamic movements.

## Competing interests

The authors declare that they have no competing interests.

## Authors' contributions

ZOK designed and implemented the feature selection, classification and control algorithm, acquired EMG data and drafted the manuscript. ZGX designed and implemented the exoskeleton prototype, performed real-time experiments and participated in manuscript preparation. CM supervised the project, contributed to discussions and analysis and participated in manuscript revisions. All authors read and approved the final manuscript.
